# Interspecific Interactions Constrain Individual Niche Divergence and Reveal Energetic Cost of Niche Expansion

**DOI:** 10.1002/ece3.74619

**Published:** 2026-11-02

**Authors:** Hunter C. Roose, James R. Junker, Jill A. Olin

**Affiliations:** ^1^ Great Lakes Research Center Michigan Technological University Houghton Michigan USA; ^2^ Department of Biological Sciences, Advanced Environmental Research Institute University of North Texas Denton Texas USA

**Keywords:** body condition, fatty acids, individual specialization, keystone niche individual, repeated measures, *Salvelinus fontinalis*

## Abstract

Individuals vary in resource use, generating trophic niche variation that shapes a population's niche width (PNW). Interspecific interactions are predicted to constrain PNW, though individual responses are often heterogeneous. The keystone niche individual (KNI) framework identifies individuals that disproportionately expand PNW by exploiting resources beyond the population's realized niche, but whether interspecific interactions limit their emergence is unknown. We tested this by comparing an allopatric and sympatric population of brook trout ( *Salvelinus fontinalis* ) and rainbow trout ( *Oncorhynchus mykiss* ). Fatty acid profiles from repeatedly sampled individuals were used to quantify individual trophic niches and their contributions to PNW. Individuals in sympatry exhibited more negative contributions to PNW (−2.70% ± 1.7%) than individuals in allopatry (−0.47% ± 1.8%) and showed a continuum of contributions rather than discrete KNI, indicating that interspecific interactions constrained niche expansion by reducing disproportionate contributions to PNW. Across both populations, individuals with greater contributions to PNW exhibited reduced trophic overlap with competitors and lower body condition, revealing energetic costs of niche expansion. These findings suggest that, in this study system, interspecific interactions can limit niche differentiation by reshaping how individuals contribute to population‐level trophic diversity, highlighting the need to evaluate the generality of these mechanisms across ecological contexts.

## Graphical abstract

Individuals differ in their contributions to population niche structure, but the ecological factors governing these differences remain unresolved. We show that interspecific interactions constrain the emergence of keystone niche individuals while exposing energetic trade‐offs associated with individuals that disproportionately expand population niche width.

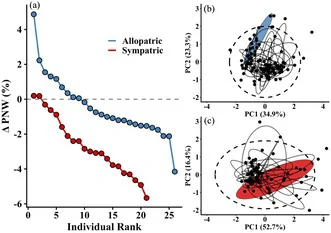

## 1 Introduction

Intraspecific variation in resource use plays a significant role in species coexistence and population persistence (Bolnick et al. 2002; Violle et al. 2012; Layman et al. 2015). The niche variation hypothesis (Van Valen 1965; West 1986) proposes that individuals can differ in resource use, a phenomenon commonly termed individual specialization, arising from intraspecific interactions, environmental heterogeneity, or behavioral constraints (Bolnick et al. 2003; Araujo et al. 2011; Liang et al. 2020; Sheppard et al. 2018). Mechanistically, the niche variation hypothesis predicts that populations exhibiting broader niche breadth should also display greater between‐individual heterogeneity, as population niche width (PNW) expansion is driven by individual divergence in resource use (Bolnick et al. 2007). In contrast, when interspecific interactions intensify and resource availability declines, PNW may contract while individuals paradoxically broaden their own resource use to buffer against reduced resource availability (Bolnick et al. 2010; Araujo et al. 2011). However, individuals rarely respond to such pressures uniformly; rather, a range of strategies to abate competitive effects may emerge (Matich et al. 2011; Manlick et al. 2021; Bolnick et al. 2010; Page and Williams 2023). These responses generate differences in niche position and raise questions about which individuals occupy positions outside the population's realized niche.

The concept of “keystone niche individuals (KNI)”, identified individuals whose resource use exerts a disproportionate influence on PNW, arising not only from broad individual niche widths, but also from where individuals are positioned within population niche space, including their eccentricity, distance from the population centroid, and the directionality of their niche ellipsoid (Garrido‐de León, Franco‐Trecu, and Costa‐Pereira 2025; Garrido‐de León, Franco‐Trecu, Naya, and Costa‐Pereira 2025). It has been suggested that KNI play a critical role in scaling individual variation to the population level by disproportionately expanding niche width and potentially enhancing the adaptive capacity of a population facing change. Despite their potential importance, the ecological and evolutionary significance of these individuals remains unclear: do they represent adaptive innovators that enhance population resilience, or subordinate competitors engaging in maladaptive behaviors that reduce condition and survival? Insight into this question comes from studies of sea otters along the Pacific coast of North America, where certain individuals in resource‐limited scenarios use tools to access otherwise unavailable prey, thereby expanding the population's realized dietary niche (Estes et al. 2003; Tinker et al. 2008). Although this strategy reflects behavioral innovation, it may also incur energetic costs associated with acquiring such prey (Law et al. 2024). Yet when resource accessibility is reduced from interspecific interactions, it is unclear if these KNI still emerge or persist.

To understand how interspecific interactions shape population niche structure, we consider how KNI influence niche diversity and expansion (Figure 1). Here, we frame niche diversity as variation in resource use between individuals (i.e., between‐individual niche width, BIC), rather than a simple continuum from specialist to generalist strategies, as individuals classified as specialists may still occupy central positions within the population niche and exert limited influence on its overall structure. Under conditions of ecological release, reductions in interspecific constraints can facilitate expansion of PNW, often through increased BIC (Bolnick et al. 2010). We propose that KNI may play a central role in this process by disproportionately driving, or reflecting, expansions in population niche space. Conversely, when interspecific interactions constrain niche expansion, the emergence or persistence of these individuals may be limited, restricting the range of resource‐use strategies expressed within a population (Figure 1). Because responses to competition or predation may vary among individuals, KNI may ultimately reflect the adaptive capacity of a population. In this way, interspecific interactions can constrain the emergence and persistence of KNI, thereby limiting niche diversity and adaptive capacity.

**FIGURE 1:**
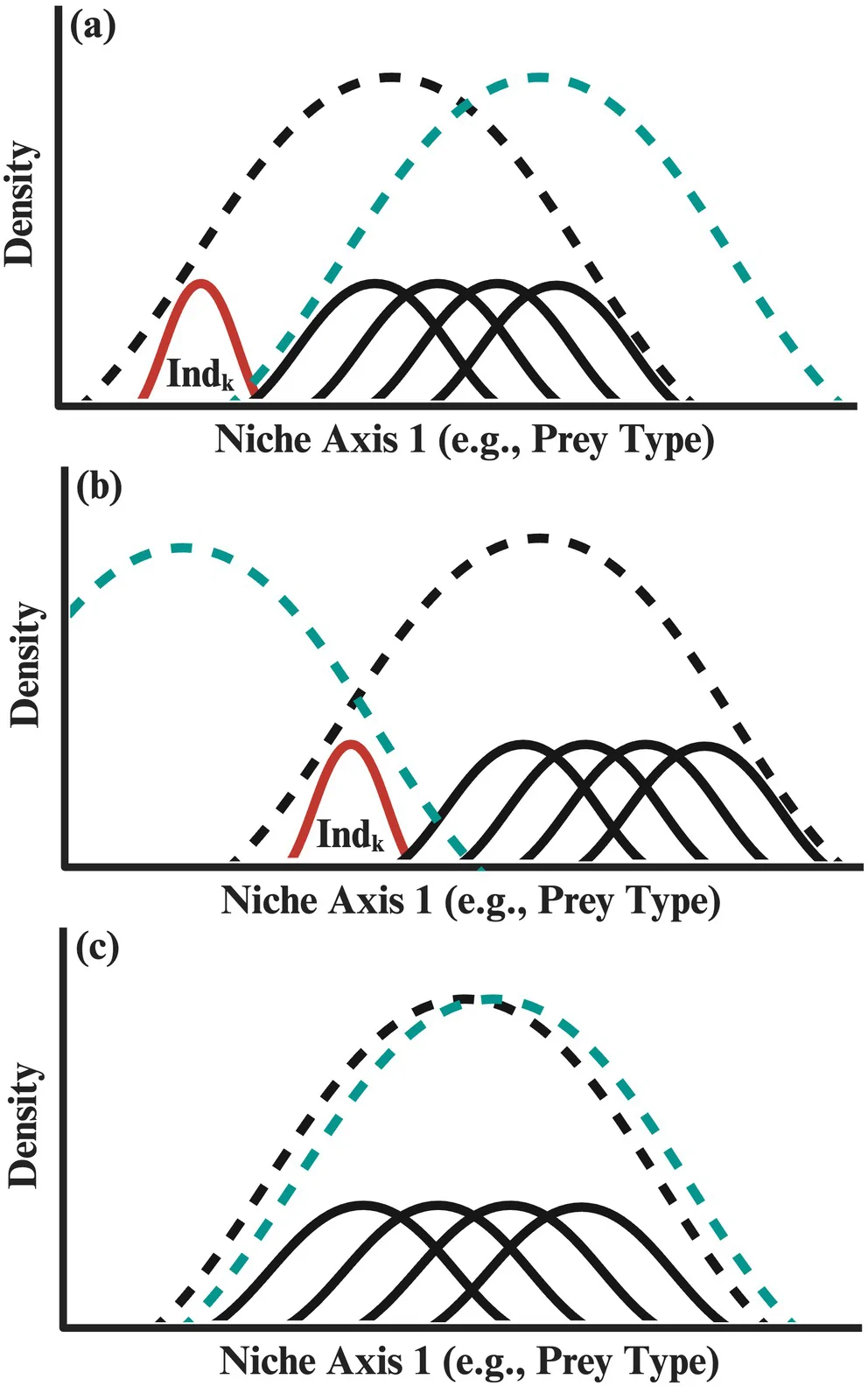
Conceptual model illustrating three potential responses of keystone niche individuals (KNI; Ind_k_) to interspecific competition. Population niches are represented by dashed line (black = reference population, teal = competitor population), while individual niches within the reference population are represented by solid lines. (a) Shift in resource use away from the competitor niche, reducing overlap. (b) Maintenance of the original niche position despite competitor presence, resulting in high overlap. (c) Absence of distinct individual differentiation.

An individual may shift their resource use away from both the population and the competitor, exhibiting character displacement (Figure 1a). We term these individuals “adaptive innovators,” as they reduce exploitative interactions by exploiting alternative resources. For example, in Darwin's finches (*Geospiza* spp.), individuals that utilize alternative seed resources under competition can reduce dietary overlap and improve survival during periods of resource limitation (Grant and Grant 2006). Such individuals effectively expand the boundaries of population niche space and, in doing so, represent KNI that provide the raw material upon which natural selection can act. Alternatively, KNI may represent maladaptive strategies, where individuals exhibit high overlap with competitors and experience reduced performance as a result (Figure 1b). For example, subordinate individuals occupying overlapping spatial niches may incur higher energetic costs and reduced access to resources (Brown 1971). These individuals may represent those that are non‐competitive among conspecifics or whose resource use reflects historical conditions that have not yet shifted. Although they may still expand the bounds of population niche space and thus be classified as KNI, they do not necessarily confer adaptive benefits and may instead reflect constrained or suboptimal strategies.

Lastly, KNI may be absent altogether (Figure 1c). Under strong interspecific competition, reduced resource availability, and increased costs of exploiting rare or novel resources may constrain niche divergence among individuals, leading to a homogenization of resource use. This outcome aligns with predictions from the niche variation hypothesis under competitive conditions, where increased ecological constraints limit individual specialization and truncate the extremes of the niche distribution (Araujo et al. 2011). As a result, individuals that would otherwise disproportionately expand PNW may fail to emerge or persist. The loss of these exploratory or extreme resource‐use strategies may inhibit niche expansion typically associated with ecological release, thereby limiting future adaptive potential by restricting the range of resource‐use strategies available for selection.

To test this concept, we use native brook trout and non‐native rainbow trout ( *Oncorhynchus mykiss* ), two widely distributed salmonids that frequently co‐occur in North American streams due to historical introductions and exhibit substantial dietary overlap (Rundio and Lindley 2008; Wilson et al. 2013; Myers et al. 2014). We evaluated a sympatric population and an allopatric population of brook trout to test how the presence of a non‐native competitor influences population niche structure and the persistence of KNI. Specifically, we ask how interspecific interactions influence population niche structure, whether KNI exist under competition, and whether occupying these positions incurs a cost in individual condition. We hypothesize that interspecific interactions restructure the population niche, leading individuals to broaden their resource use, thereby (1) reducing the occurrence or magnitude of KNI and (2) decreasing condition among individuals exploiting resources outside the population core niche. Finally, we evaluate whether an individual's contribution to PNW (i.e., keystoneness) is related to its degree of overlap with a competitor. If KNI represent adaptive strategies that reduce competitive interactions, we expect individuals with greater contributions to PNW to exhibit lower overlap with competitors. Alternatively, if KNI reflects maladaptive or constrained strategies, we expect greater niche contribution to be associated with higher overlap with the competitor. This framework links individual‐level variation to population‐level consequences, providing a mechanistic basis for understanding how interspecific interactions shape ecological outcomes.

## 2 Materials and Methods

### 2.1 Study System and Sample Collection

The study was conducted in the Salmon‐Trout River watershed, located in Michigan's Upper Peninsula within the North American temperate region (Figure 2). The third‐order Salmon‐Trout River drains a watershed dominated by deciduous forest (53%), mixed forest (24%), woody wetlands (16%), and developed land (2%). The streams feature shallow riffles with gravel and cobble substrates, interspersed with deeper pools and runs characterized by sandy substrates that accumulate detrital material. Since the 1930s, brook and rainbow trout have co‐occurred in the Salmon‐Trout River (hereafter, sympatric), with no fish stocking since the 1960s (Figure 2). The Salmon‐Trout River had a mean wetted width of 9.6 ± 1.9 m, depth of 0.36 ± 0.18 m, canopy cover of 50.4% ± 30.4%, and a water temperature of 11.9°C ± 2.3°C. In contrast, the second‐order Obenhoff Creek, a tributary inhabited solely by brook trout (hereafter, allopatric; Figure 2), was narrower (wetted width = 4.5 ± 2.0 m) but had a similar depth (0.41 ± 0.23 m). Obenhoff Creek also had a greater canopy cover (75.9% ± 36.8%) and a higher water temperature (15.6°C ± 4.3°C).

**FIGURE 2:**
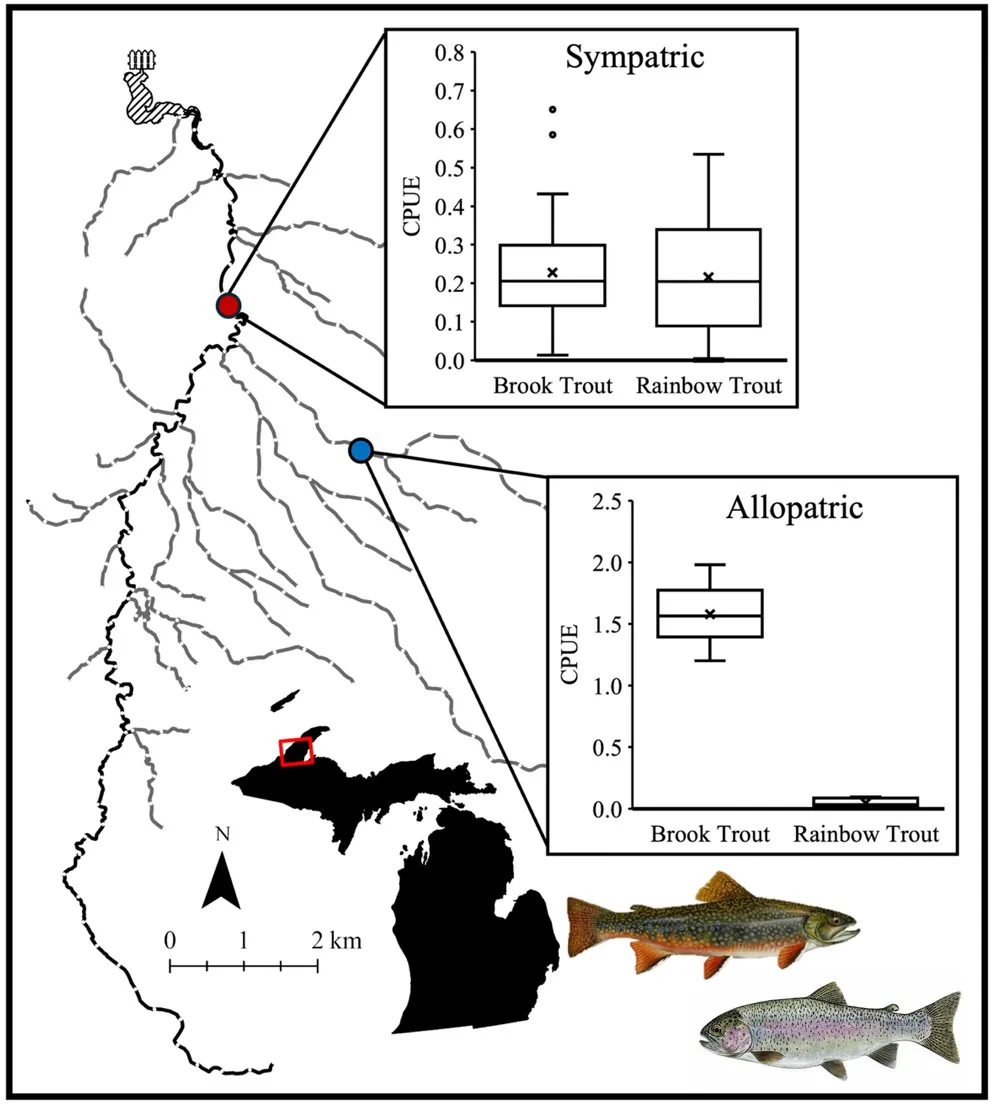
Study sites in Michigan's Upper Peninsula, USA, with catch‐per‐unit‐effort (CPUE) for brook and rainbow trout shown for both rivers (Roose, unpublished data).

Fish were collected from both streams monthly from May to September of 2023–2025 using backpack electrofishing (ABP‐2; ETS Electrofishing Systems, Madison, WI, USA). All individuals > 110 mm total length (TL) were anesthetized with Aqui‐S 20E (20 mg L^−1^), measured for TL and body mass (A&D HL‐3000WP analytical balance), and implanted with a 12 mm PIT tag (APT12, Biomark, Boise, ID, USA; VodaIQ, Ferry, ID, USA) near the pectoral fin. Whole blood (0.1–0.3 mL) was drawn from the caudal vein using a 23G needle and 1 mL luer‐lock syringe, transferred into 4 mL sodium heparin tubes and kept on ice in the field. Fish recovered in an aerated bath and then released back into the stream.

### 2.2 Fatty Acid Analysis

Blood samples were freeze‐dried for 48 h and weighed (20 mg) into a 10 mL test tube. Total lipids were extracted, and fatty acid methyl esters (FAMEs) were derived using a one‐step acid‐catalyzed transesterification method modified from Indarti et al. (2005) and Richoux and Ndhlovu (2014). Chloroform containing 0.01% butylated hydroxytoluene (2 mL) was added to each sample, followed by 2 mL of sulfuric acid and methanol (0.3:1.7). To assess recovery efficiency, 10 μL of internal standard solution (~5 mg C19:0 dissolved in 5 mL chloroform) was added and samples were heated at 100°C for 30 min. Ultra‐pure Milli‐Q water was added to quench the reaction, and phases were separated by centrifugation. The lower organic phase was purified, dried under nitrogen, and FAMEs were re‐suspended in hexane prior to gas chromatography using helium as the carrier gas (Happel et al. 2017). The relative abundance of each fatty acid was calculated as its proportion of the total fatty acid pool (% TFA).

### 2.3 Data Analysis

All statistical analyses were performed in R (R development Core Team 2025) within the RStudio interface (Posit Team 2026). Only brook trout with ≥ 3 blood draws were analyzed.

### 2.4 Fatty Acid Niche

Dietary fatty acids (FA) are powerful biochemical tracers of resource use because many FA are assimilated with limited modification and therefore reflect basal resource pathways and trophic interactions (Parrish et al. 2000; Iverson et al. 2004; Torre‐Ruiz et al. 2007). Individual fatty acids can serve as markers of specific prey types or basal resources. For example, eicosapentaenoic acid (EPA, 20:5*n* − 3) is commonly associated with aquatic‐derived energy pathways in stream food webs, whereas omega‐6 fatty acids and C18 precursors such as α‐linolenic acid (ALA, 18:3*n* − 3) and linoleic acid (LIN, 18:2*n* − 6) are frequently associated with allochthonous inputs from terrestrial riparian environments (Twining et al. 2017). Within streams, macroinvertebrates can be grouped into distinct foraging functional groups that exhibit characteristic fatty‐acid profiles; for example, grazers that feed primarily on epilithic periphyton and algae are typically enriched in EPA and bacterial fatty acids, such as 15:0, 17:0, and 18:1*n* − 7, whereas shredders that specialize on leaf litter exhibit higher proportions of ALA and LIN, reflecting greater contributions from terrestrial carbon sources (Guo et al. 2018). Because organisms integrate dietary inputs over time through tissue assimilation, FA profiles represent a temporally integrated signature of resource use. Specifically, FA derived from the blood are particularly useful for examining shorter‐term (~2–3 weeks) dietary assimilation (Käkelä et al. 2009; Tierney et al. 2008). We define the FA niche of an individual as the multivariate hypervolume described by the relative composition of FA in its blood, where each FA represents a distinct axis of resource use. In this framework, the position and shape of the hypervolume capture both the diversity of resources used and their relative allocation by the individual. In addition to reflecting resource use, variation in FA composition can provide insight into the nutritional condition or energetic status of an individual, linking dietary intake to physiological state.

### 2.5 Keystone Niche Individuals

Fatty acids contributing > 1% to the total FA profile were logit‐transformed to meet assumptions of normality (Greenacre 2021) and reduced to two principal components (PC1 and PC2) using principal component analysis (PCA) implemented in the R package ‘vegan’ (Oksanen et al. 2026).

Population variance structure was quantified using bivariate generalized linear mixed models (GLMMs) to estimate PNW (as a proxy for total niche width). These models integrate variation across multiple niche dimensions, providing a robust estimate of total population variance (Ingram et al. 2018). Separate bivariate models were fit for each stream with PC1 and PC2 specified as response variables and individual included as a random effect using the ‘MCMCglmm’ package (Hadfield 2010). Weakly informative priors were used for both random‐effect and residual variance–covariance matrices, and models were run for 50,000 iterations following a burn‐in of 10,000 iterations with a thinning interval of 50. Within this framework, the residual (R) variance–covariance structure represents the within‐individual variance (WIC), while the random‐effect (G) structure represents BIC (Ingram et al. 2018). PNW was calculated as the sum of WIC and BIC (Bolnick et al. 2003).

To estimate an individual's contribution to PNW, we quantified the change in PNW following the sequential removal of individuals from the population. Specifically, we compared PNW estimated with all individuals present (PNW_*b*_) to PNW recalculated after removing a focal individual (PNW_*b*−1_) (Garrido‐de León, Franco‐Trecu, Naya, and Costa‐Pereira 2025). The resulting ΔPNW reflects the combined change in WIC (ΔWIC) and BIC (ΔBIC). This removal‐based approach allows for the identification of individuals whose presence disproportionately structures population‐level niche breadth. Rather than applying a fixed threshold, individuals were evaluated based on their relative influence on PNW, with those producing the largest reductions upon removal interpreted as exerting the strongest influence on population niche structure (i.e., candidate KNI). Conversely, if removal of an individual increased PNW, they exhibited a negative ΔPNW and were interpreted as occupying central positions within the population niche, with their presence effectively stabilizing or constraining BIC.

### 2.6 Quantifying Niche Properties

To characterize how individuals differ in their trophic niches within populations, we quantified four complementary niche properties derived from each population's FA niche. These metrics capture distinct aspects of individual niche expression, including niche breadth, position, shape, and orientation relative to the population (Garrido‐de León, Franco‐Trecu, Naya, and Costa‐Pereira 2025). Together, they provide insight into why individuals may exert a disproportionate influence on PNW.

Individuals exploiting a broader diversity of resources are expected to occupy larger regions of niche space, whereas specialists exhibit narrower niches. We quantified individual niche width using the standard ellipse area (SEAc) estimated from each fish's PC1–PC2 scores using the ‘SIBER’ package in R (Jackson et al. 2011). Total niche area (TA) was calculated as the convex hull encompassing all individuals within each population. To contextualize individual breadth relative to population niche size, we calculated the Relative Individual Niche Index (niche breadth) = SEAc_individual_/TA_population_. Niche breadth values were min–max standardized (0–1) and square‐root transformed, where higher values indicating a greater proportional occupation of population niche space (e.g., Sheppard et al. 2018; Manlick et al. 2021). Individual niche position was quantified as the Euclidean distance between an individual's niche centroid and the population centroid (Newsome et al. 2012). Individuals located near the population centroid have similar FA profiles to the population average and can be interpreted as primarily exploiting commonly used resources. In contrast, individuals positioned farther from the centroid reflect distinct or uncommon resource use. Importantly, individuals with small niche widths may still exert a disproportionate influence on PNW if their niche position extends the population into previously unoccupied regions of FA space. Individual niche orientation was calculated as the angle between the major axis of an individual's standard ellipse and PC1. The orientation angle (θ) was calculated from the eigenvector (*v* = *v*_1_, *v*_2_) associated with the largest eigenvalue of the covariance matrix of PC1 and PC2 scores, which defines the major axis of the ellipse. Orientation relative to PC1 was calculated as *θ* = arctan (*v*_2_/*v*₁). Relative niche orientation was then calculated as the angular difference between an individual's ellipse orientation and the population ellipse orientation and may reflect alternative foraging strategies or trophic pathways not captured by niche width or position alone. We quantified ellipse eccentricity from this same covariance matrix for each individual to describe how variation is distributed across axes. Eigenvalues of the covariance matrix (*λ*_1_ ≥ *λ*_2_) represent the variance along the major and minor ellipse axes, respectively. Eccentricity was calculated as $e=\sqrt{1-\left(\lambda_{2}/\lambda_{1}\right)}$. High eccentricity indicates that niche variation is concentrated along a single dominant fatty acid gradient, consistent with specialization along one trophic pathway. Low eccentricity indicates more evenly distributed variation across axes, suggesting multidimensional resource integration. Eccentricity therefore captures whether individuals differ primarily along one biochemical pathway or across multiple resource gradients simultaneously.

### 2.7 Estimating Intra and Interspecific Overlap

To quantify dietary overlap, we used a proportional similarity (PS) index, which measures multivariate overlap in resource use and ranges from 0 (no overlap) to 1 (complete overlap), following Bolnick et al. (2002). The PS index was calculated as:

$$PS_{i}=1-\frac{1}{2}\sum_{j}|p_{\mathrm{ij}}-q_{j}|$$

where $p_{\mathrm{ij}}$ is the proportion of FA *j* in individual i, and $q_{j}$ is the proportional representation of FA *j* in the reference population. For interspecific overlap, we calculated PS between each brook trout observation (i.e., individual blood draw) and the mean FA profile of the rainbow trout population (Table S9). For intraspecific overlap, we calculated PS between each individual observation and the mean FA profile of the brook trout population. PS values were averaged across observations to obtain individual‐level estimates. To evaluate whether individual niche contribution was related to overlap, we used linear regression to model the relationship between individual PS values and their corresponding contribution estimates.

### 2.8 Individual Condition

Fatty acid composition can provide insight into individual body condition, with implications for growth, reproduction, and overall performance (Twining et al. 2016; Scharnweber and Gårdmark 2020). Specific FA including EPA and DHA, as well as broader groupings including polyunsaturated FA and saturated FA, are often associated with variation in nutritional quality and physiological condition. We evaluated relationships between these FA (both individual compounds and summed groups) and each fish's contribution to PNW to assess potential tradeoffs between niche contribution and condition. In addition to FA‐based metrics, we quantified individual body condition using a residual condition index derived from stream‐specific length–mass relationships following Le Cren (1951). For each stream, we fit a regression between body mass and length and estimated expected mass for a given length. Individual condition was then calculated as the deviation between observed and expected mass, such that individuals with positive values were in above‐average condition and those with negative values were in below‐average condition. This approach accounts for stream‐specific growth differences, allowing comparison among individuals within and across systems. We then related this condition index to individual keystoneness to further evaluate whether individuals that disproportionately contribute to PNW incur a cost in body condition.

### 2.9 Statistical Analysis

The contribution to PNW was compared between streams using analysis of variance (ANOVA). The effect of niche properties on contribution between streams was tested using linear models. The distribution of the response variable (∆PNW) was evaluated using the ‘fitdistrplus’ package (Delignette‐Muller and Dutang 2015), and Gaussian models were found to provide an adequate fit based on the Akaike information criterion (AIC). Model assumptions were verified using Shapiro–Wilk and Breusch‐Pagan tests. Full models included niche breadth, niche position, eccentricity, and relative orientation, and were simplified using AIC‐based stepwise selection (ΔAIC < 2; Crawley 2012). The mean proportion of individual fatty acids was examined in relation to ∆PNW to assess whether an individual's contribution to PNW was influenced by these essential biomolecules. Linear models were used to test associations between metrics of condition and ∆PNW.

## 3 Results

Forty‐seven individuals met the criteria for three sampling events (allopatric: *n* = 26; sympatric: *n* = 21), representing 176 total sampling events (range 3–8 times per individual; Tables S1–S4). The bivariate mixed‐effects model estimated larger PNW in sympatric (posterior mean = 0.95, 95% credible interval = 0.73–1.30) compared to the allopatric (0.70, 0.56–0.90) population. This difference was driven by greater WIC in sympatry (0.52, 0.40–0.68) compared to allopatry (0.35, 0.29–0.43). The BIC was slightly higher in sympatry (0.44, 0.26–0.72) than in allopatry (0.35, 0.22–0.55), although credible intervals overlapped substantially.

The contribution of individuals to PNW differed significantly between systems. Individuals in sympatry exhibited a more negative mean contribution to PNW (−2.70% ± 1.7%) compared to allopatry (−0.47% ± 1.8%; ANOVA: *F*_1,45_ = 19.54, *p* < 0.001) (Figure 3a). Individuals with values above 0 act to expand PNW, whereas those below 0 constrain PNW (Garrido‐de León, Franco‐Trecu, Naya, and Costa‐Pereira 2025). Individuals exceeding 0 may therefore be considered candidates for keystone niche individuals. A diverse pattern of contribution was more evident in allopatry, where 9 of 26 individuals (34.6%) exhibited positive contributions to PNW. In contrast, only 2 of 21 individuals (9.5%) in sympatry were above 0. Moreover, the maximum individual contribution in allopatry reached 4.87%, whereas the highest value observed in sympatry was only 0.20%.

**FIGURE 3:**
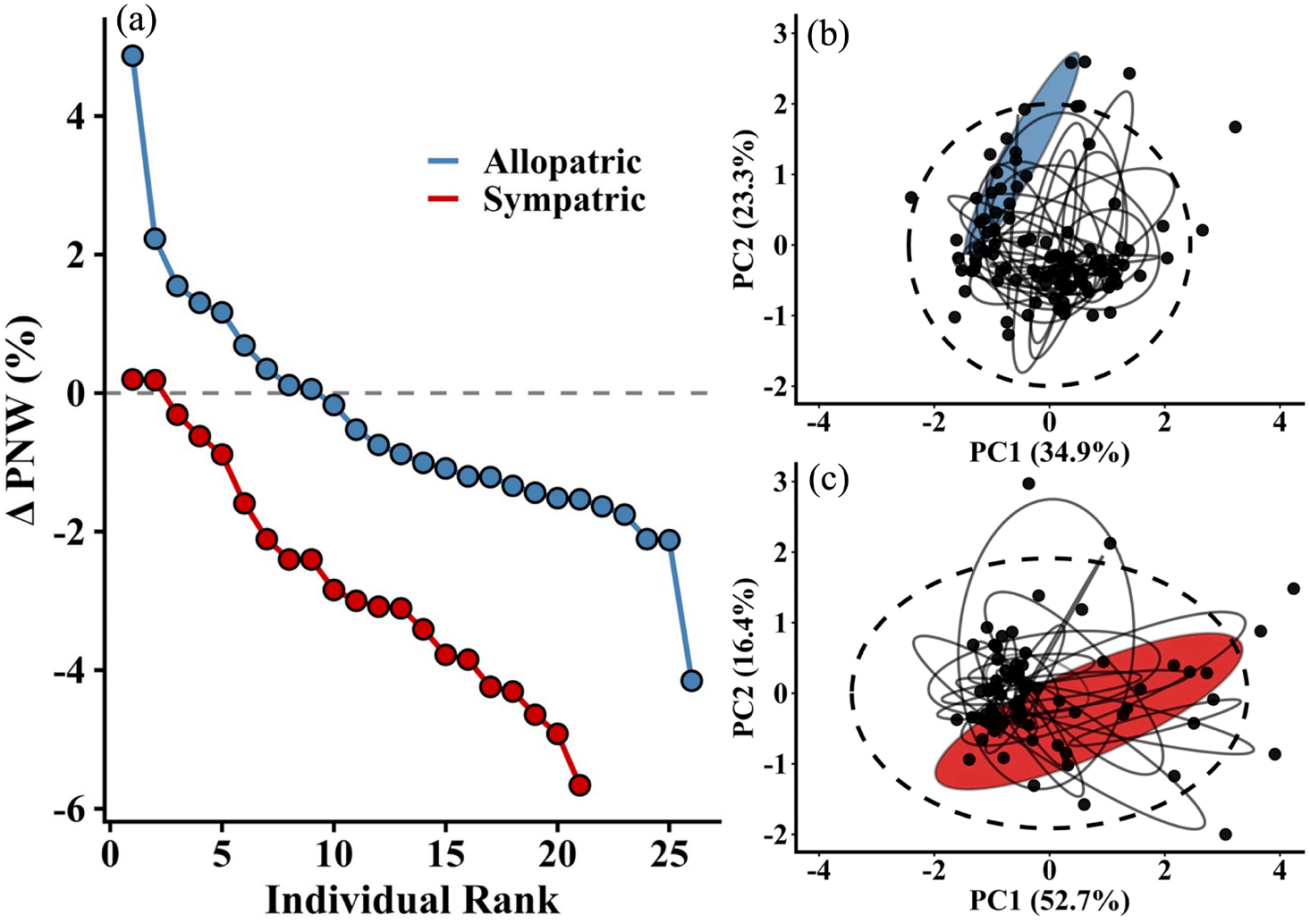
(a) Individual contributions to population niche width for brook trout by stream. Individuals are ranked by their effect on population niche width (PNW), calculated as the change in population niche area following individual removal (PNW_*b*_ − PNW_*b‐i*_). Positive and negative values indicate individuals that expand and constrain population niche space, respectively. Principal component analysis of FA profiles for brook trout in allopatry (b) and sympatry (c). Solid ellipses represent individual‐level variance, and dashed ellipses represent population‐level variance of FA niche space. Shaded ellipses highlight KNI.

Niche position and niche breadth influenced the contribution of individuals to PNW, although the effects were not consistent across systems. For allopatry, the most parsimonious model identified niche position (distance to centroid) as the strongest predictor of ∆PNW (*β* = 0.024, *p* < 0.01) (Figure 4; Table S5). Niche breadth, eccentricity, and relative orientation had no significant effect. In contrast, for sympatry, the best model retained niche breadth (*β* = −0.03, *p* < 0.05) and niche position (*β* = 0.02, *p* < 0.05) as significant predictors of ∆PNW, while relative orientation and eccentricity were non‐significant (Figure 4; Table S6).

**FIGURE 4:**
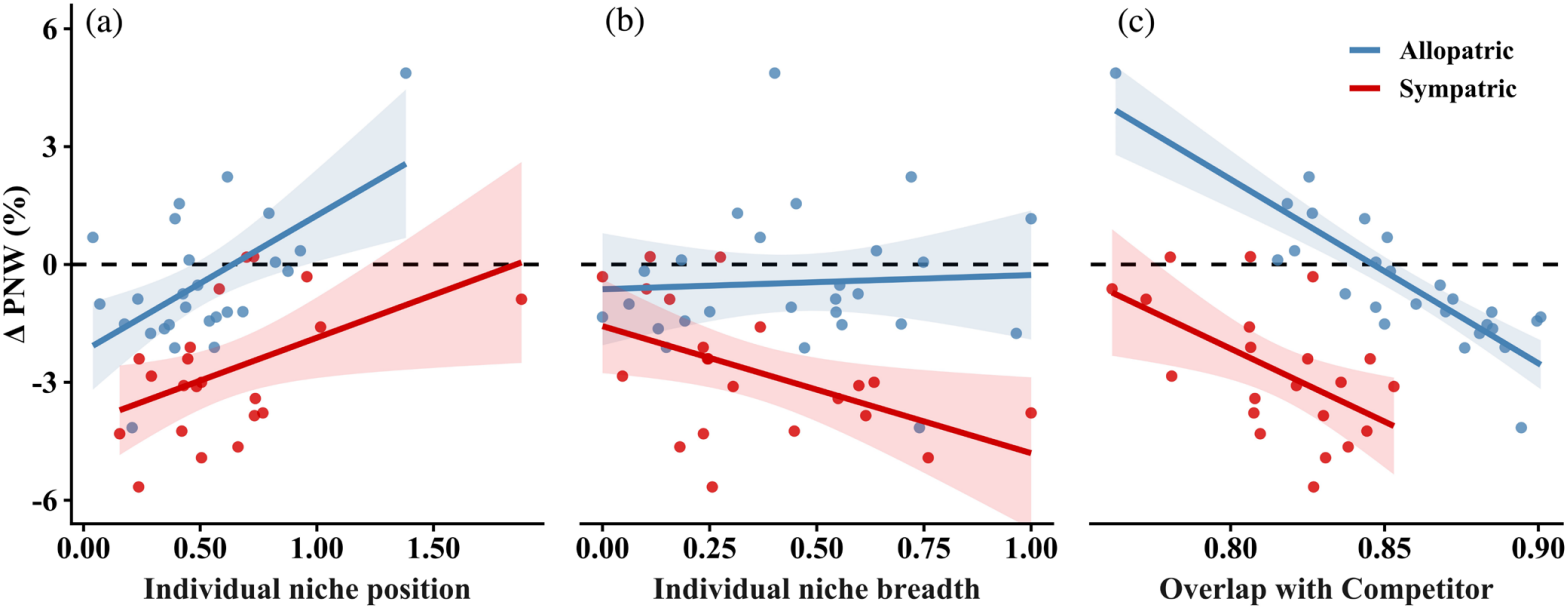
Relationship (with 95% confidence intervals) between niche properties and individual contribution to population niche width (PNW). (a) Effect of individual niche position, measured as the distance of an individual's niche from the population centroid. (b) Individual niche breadth as relative individual niche index, scaled from 0 to 1 (0 = specialist; 1 = generalist). (c) Degree of overlap between competitors as proportional similarity index (0 = no overlap; 1 = complete overlap).

In allopatry, the KNI had a PS of 0.763 ± 0.088 relative to the population, whereas the lowest‐ranking individual had a PS of 0.894 ± 0.018. Across all conspecifics, PS ranged from 0.763 to 0.901. Linear regression indicated that individuals with higher overlap contributed less to the population‐level niche, and this relationship was strong in allopatry (slope = −2.291, *p* < 0.00001, *R* ^2^ = 0.75) (Figure 4). In sympatry, the highest‐ranking ‘keystone’ individual had a PS of 0.806 ± 0.055 relative to rainbow trout, whereas the lowest‐ranking individual had a PS of 0.827 ± 0.044. Across individuals, overlap values ranged from 0.761 to 0.853. The negative relationship between individual PS and niche contribution was also present in sympatry, though weaker (slope = −0.845, *p* = 0.0104, *R* ^2^ = 0.30) (Figure 4).

Linear regression analyses revealed that individual contributions to PNW were associated with variation in FA profiles as well as body condition (Figure 5; Tables S7 and S8). Fatty acid patterns were broadly consistent across streams: in both the sympatric and allopatric contexts, individuals enriched in saturated FA and monounsaturated FA contributed more to PNW, whereas individuals enriched in polyunsaturated FA, including 18:3n‐3 (ALA), 20:5*n* − 3 (EPA), 22:5*n* − 3, and 22:6*n* − 3 (DHA), contributed less. In contrast, body condition differed between streams: in allopatry, higher condition factor was associated with reduced individual contribution to PNW (*β* = −10.78, *p* = 0.044, *R* ^2^ = 0.16), whereas in sympatry there was no detectable relationship (*β* = −1.57, *p* = 0.81, *R* ^2^ = 0.003).

**FIGURE 5:**
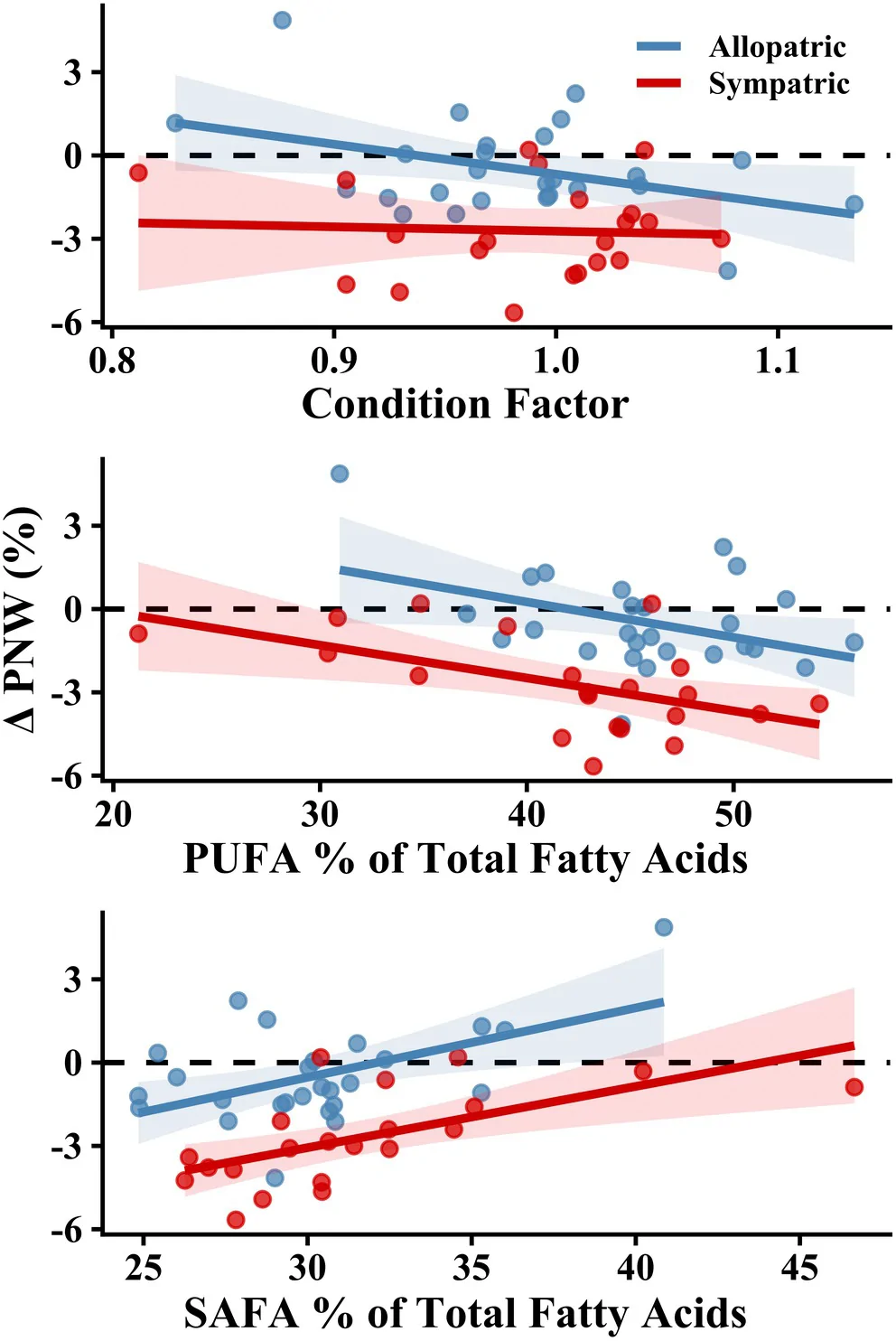
Relationship (with 95% confidence interval) between individual contribution to population niche width (PNW) and average body condition, polyunsaturated fatty acid (PUFA) composition, and saturated fatty acid (SAFA) composition.

## 4 Discussion

We observed contrasting patterns of niche structure and individual contributions to PNW between the allopatric and sympatric populations. In the sympatric population, we did not detect discrete KNI; instead, most individuals constrained PNW. In contrast, the allopatric population exhibited greater niche diversity, with individuals both expanding and constraining PNW, indicating more heterogeneous resource use among individuals. This pattern is consistent with the higher WIC observed in the sympatric population, reflecting greater individual generalization and increased overlap in resource use. Mechanisms underlying these patterns also differed between systems. In both populations, individuals farther from the population centroid exerted stronger effects on PNW, consistent with the use of distinct resources. However, in sympatry these individuals were specialists with low niche breadth, whereas in allopatry high‐contributing individuals included both generalists and specialists. Individuals contributing most to PNW also showed reduced overlap with competitors in both populations, suggesting that niche differentiation may alleviate competition. FA composition was consistently associated with individual contributions to PNW, suggesting energetic or physiological costs of exploiting resources outside the population core niche, whereas condition factor was context‐dependent and influenced PNW only in the allopatric system. Collectively, these results indicate that competitive interactions structure individual contributions to PNW and show energetic costs associated with niche expansion, providing context for the identity and expression of the KNI.

Intraspecific interactions predominantly structured the population niche in the allopatric system, supporting the niche variation hypothesis, as competition among conspecifics promotes niche complementarity and elevated niche diversity. Consistent with this mechanism, Svanbäck and Bolnick (2007) showed that increased intraspecific competition in *Gasterosteus aculeatus* drives individual specialization by selecting for reduced resource overlap among individuals (i.e., increase in BIC). We found that KNI were located farther from the population centroid and exhibited reduced overlap with conspecifics. While such individuals are often interpreted as adaptive innovators, our results suggest an alternative explanation. These individuals exhibited lower condition based on both body condition and FA composition, suggesting they may be competitively inferior and consequently forced to exploit suboptimal resources. This interpretation aligns with ecological theory predicting that competitively inferior individuals shift to alternative resources when displaced from the population niche core. It is important to note that other fish species (e.g., *Cottidae* spp.) are present in these systems and may influence the observed pattern. However, these species are native to these streams and have coevolved with focal taxa, such that competitive interactions are likely weaker than those imposed by non‐native species such as rainbow trout. As a result, their influence on niche structure is likely comparatively limited, as coevolved communities are expected to exhibit more stable niche partitioning (Wilson 1988), allowing between‐individual variation to persist.

Interspecific interactions in the sympatric system appeared to constrain population niche structure by promoting resource use homogenization among conspecifics, as evidenced by an increase in WIC. However, PNW was larger in the sympatric than in the allopatric system, contrary to expectations based on previous studies (Bolnick et al. 2010; Araujo et al. 2011). This pattern may reflect differences in resource availability, as greater prey diversity in downstream environments can support a broader population niche. Despite this, elevated WIC indicates greater individual generalism and is consistent with the hypothesis that interspecific interactions can reshape niche structure by reducing differentiation among individuals, as increases in PNW are often driven by increases in BIC (Bolnick et al. 2003). However, differences in environmental context between the study systems, including abiotic conditions and population density, may also contribute to the observed patterns, making it difficult to disentangle the effects of interspecific interactions from other sources of variation. Overall, these results suggest that resource availability may influence PNW, while the increase in WIC is consistent with the hypothesis that interspecific interactions influence how resource use is distributed among individuals. Further comparisons across replicated allopatric and sympatric systems are needed to evaluate the relative contributions of these factors.

No single individual disproportionately influenced the population niche in the sympatric system, suggesting a continuum of keystone niche influence (“keystone gradient”) rather than discrete keystone niche individuals. This result refines the KNI framework by indicating that the expression of keystoneness may depend on ecological context, particularly the presence of competitors, and may reflect populations that have adapted to persistent interspecific interactions. Nevertheless, individual traits remained predictive of niche influence, with distance to the population centroid and niche breadth strongly associated with contributions to PNW. Moreover, keystoneness was negatively related to overlap with competitors, suggesting that individuals with greater influence on population niche structure reduce competition through niche differentiation. To this point, individuals exhibiting more generalist strategies in sympatry showed the highest overlap with rainbow trout, indicating that generalization may function as a compensatory response to resource limitation rather than a strategy of niche expansion. While our results provide support for context‐dependent expression of keystoneness within this study system, testing this framework across replicated brook trout‐rainbow trout systems and additional species assemblages will be important for evaluating the generality of these patterns. Incorporating additional niche axes such as prey size may reveal hidden dimensions of individual specialization not captured by FA profiles. Indeed, recent work (e.g., Baker et al. 2022) shows that prey size diversity can be a primary driver of increased BIC in brook trout populations. Similarly, spatial resource use may represent another key axis along which individuals differ, suggesting that integrating movement ecology with trophic analyses could improve identification of KNI.

Importantly, these results highlight a key nuance in the interpretation of KNI, which may be assumed to advantage the individuals occupying them. Instead, individuals that expand the population niche do so at a cost, as indicated by reduced condition. Reduced condition associated with the use of distinct resources is consistent with predictions from optimal foraging theory, which posits that individuals maximize energetic returns by selecting resources within an optimal range (MacArthur and Pianka 1966). Deviation from this range, potentially reflected by resource use outside the population core, may result in lower energetic efficiency and, consequently, reduced body condition. A key strength of this study is the use of FA composition to infer nutritional condition, as reduced levels of essential FA are known to have important fitness consequences, including decreased growth rates, reproductive capacity, and cognitive performance (Twining et al. 2016; Scharnweber and Gårdmark 2020; Závorka et al. 2022). One limitation of this approach is that FA composition measured from blood reflects both dietary intake and endogenous physiological regulation, including the selective mobilization, synthesis, and incorporation of FA associated with growth, maintenance, and reproduction. Consequently, blood fatty acid profiles may also vary with sex and reproductive state. However, despite these physiological influences, blood FA profiles retain proportional relationships with dietary sources, supporting their use as indicators of resource use (Käkelä et al. 2009; Tierney et al. 2008). Therefore, we analyzed fatty acids as proportions of total fatty acids rather than absolute concentrations to reduce the influence of differences in total fatty acid abundance. Furthermore, when examining relationships between summed FA values and individual keystoneness, we used mean values across repeated measurements for each individual to reduce the influence of temporal variation in FA profiles associated with physiological state. Additionally, while individuals occupying keystone niche positions may exhibit reduced condition at a given time, these roles are likely context‐dependent, and individuals may experience improved condition under different environmental conditions, suggesting that keystoneness is dynamic rather than fixed.

Niche diversity driven by KNI has direct implications for the adaptive capacity of populations. The loss of such individuals may therefore reduce the range of phenotypes available for niche expansion under changing environmental conditions (Schindler et al. 2015). Beyond evolutionary consequences, reduced BIC may also decrease population stability and ecosystem functioning through mechanisms such as the portfolio effect, whereby greater heterogeneity buffers temporal variability in performance. Consistent with this, increased individual heterogeneity has been shown to enhance productivity in systems such as salmon (Griffiths and Armstrong 2001) and fire‐bellied toads (Jasieński 1988), likely through more efficient resource partitioning and niche complementarity that facilitates energy transfer across trophic pathways. From an ecosystem functioning perspective, KNI may therefore play a disproportionate role in maintaining multiple energy pathways within food webs. Their use of distinct resources may enhance the diversification of energy flow, whereas their loss could promote trophic homogenization and reduce the efficiency and dimensionality of energy transfer through the system. Overall, the KNI framework provides a useful perspective for understanding how individual variation structures population dynamics, ecosystem functioning, and associated environmental processes, and highlights important avenues for future research across ecological systems.

Keystone niche individuals differed in their expression between allopatric and sympatric systems. Greater niche diversity and evidence of KNI were observed in allopatry, where niche complementarity likely reduced intraspecific competition. In this context, these individuals may represent competitively inferior individuals forced to exploit alternative resources. In sympatry, individuals largely constrained the population niche, and although no single individual was identified as KNI, the presence of a gradient suggests that such effects may persist in a more diffuse form. These patterns highlight strong context dependency in the expression of keystone niche individuals. Under intraspecific competition, they may reflect competitive displacement, whereas under interspecific competition, they may instead function as adaptive innovators that reduce niche overlap. Experimental manipulation of competitor density may further clarify the context dependency of these interactions, as different competitors can generate distinct ecological outcomes (Bolnick et al. 2010). Ultimately, the loss or weakening of these individuals may reduce population resilience and limit the capacity for niche expansion under environmental change.

## Author Contributions

**Hunter C. Roose:** conceptualization (lead), data curation (lead), formal analysis (lead), funding acquisition (supporting), investigation (lead), methodology (lead), project administration (supporting), resources (supporting), visualization (lead), writing – original draft (lead), writing – review and editing (lead). **James R. Junker:** formal analysis (supporting), methodology (supporting), software (supporting), validation (supporting), visualization (supporting), writing – review and editing (supporting). **Jill A. Olin:** conceptualization (supporting), data curation (supporting), formal analysis (supporting), funding acquisition (lead), investigation (supporting), methodology (supporting), project administration (lead), resources (lead), supervision (lead), validation (supporting), visualization (supporting), writing – original draft (supporting), writing – review and editing (equal).

## Funding

Funding was provided by Michigan Technological University's Research Excellence Fund (JAO) (REF‐RS 25‐0198; JAO) and Great Lakes Research Center's Student Research Grant program (HCR).

## Ethics Statement

Fish were sampled under state and federal permits (FSCP01112023165727), and all procedures were approved by the Institutional Animal Care and Use Committee (2029815) in accordance with National Institutes of Health guidelines.

## Conflicts of Interest

The authors declare no conflicts of interest.

## Supporting information


**Table S1.** Metadata of individuals captured in Salmon‐Trout River, total length (TL) and mass (g) expressed as: (mean ± SD).
**Table S2.** Metadata of individuals captured in Obenhoff Creek, total length (TL) and mass (g) expressed as: (mean ± SD).
**Table S3.** Fatty acid composition of each individual (mean ± standard deviation) in the Salmon‐Trout River.
**Table S4.** Fatty acid composition of each individual (mean ± standard deviation) in the Obenhoff Creek.
**Table S5.** Linear model results from stepwise comparison for Obenhoff Creek, with change in total niche width (ΔTNW) as the response variable.
**Table S6.** Linear model results from stepwise comparison for Salmon‐Trout River, with change in total niche width (ΔTNW) as the response variable.
**Table S7.** Linear regression results of the contribution to TNW of an individual and the average fatty acid content for Salmon‐Trout River. Rows in bold indicate significant relationships.
**Table S8.** Linear regression results of the contribution to TNW of an individual and the average fatty acid content for Obenhoff Creek. Rows in bold indicate significant relationships.
**Table S9.** Population data of Salmon‐Trout River and Obenhoff Creek, total length (TL) and mass (g) and fatty acids (percent of total fatty acids) expressed as: (mean ± SD).

## Data Availability

All the required data are uploaded as supplementary material.
