# Supplementary material for: Interspecific Interactions Constrain Individual Niche Divergence and Reveal Energetic Cost of Niche Expansion
Source: Ecol Evol. 2026 Nov 2;16(11):e74619. doi: 10.1002/ece3.74619 (PMC13728540; doi:10.1002/ece3.74619)
Supplement: Supplementary file 1 [file ECE3-16-e74619-s001.docx]

**SUPPLEMENTAL**

| **Table S1**. Metadata of individuals captured in Salmon-Trout River, total length (TL) and mass (g) expressed as: (mean ± SD) | | | | |
| --- | --- | --- | --- | --- |
| **Individual ID** | **n** | **TL (mm)** | **Mass (g)** | **Sampling Events (Month - Year)** |
| 900251000082552 | 3 | 191.0 ± 32.4 | 74.7 ± 42.2 | (July - 2024, May - 2025, July - 2025) |
| 900251000082554 | 3 | 170.3 ± 31.6 | 52.7 ± 34.1 | (July - 2024, Sep - 2024, June - 2025) |
| 900251000082571 | 4 | 278.0 ± 13.6 | 206.5 ± 38.4 | (July - 2024, May - 2025, July - 2025, Aug - 2025) |
| 900251000082645 | 3 | 130.0 ± 2.8 | 20.0 ± 1.4 | (June - 2024, Aug - 2024, Sep - 2024) |
| 900251000082666 | 3 | 248.3 ± 18.3 | 152.7 ± 29.3 | (June - 2024, July - 2024, Sep - 2024) |
| 900251000082822 | 3 | 203.0 ± 19.7 | 83.7 ± 30.7 | (May - 2025, June - 2025, Sep - 2025) |
| 900251000082854 | 3 | 142.3 ± 20.5 | 26.3 ± 9.5 | (May - 2025, June - 2025, Sep - 2025) |
| 900251000082887 | 3 | 191.0 ± 5.0 | 68.0 ± 3.0 | (May - 2025, June - 2025, July - 2025) |
| 989001044609756 | 4 | 180.5 ± 17.5 | 53.2 ± 17.2 | (July - 2024, Sep - 2024, June - 2025, Aug - 2025) |
| 989001044609767 | 3 | 197.7 ± 19.8 | 66.7 ± 13.3 | (May - 2024, June - 2024, Aug - 2024) |
| 989001044609784 | 3 | 130.3 ± 3.2 | 19.0 ± 2.0 | (July - 2024, Aug - 2024, Sep - 2024) |
| 989001044609818 | 6 | 216.5 ± 27.4 | 100.0 ± 36.8 | (May - 2024, July - 2024, Aug - 2024, Sep - 2024, May - 2025, June - 2025) |
| 989001044609841 | 4 | 211.0 ± 18.6 | 99.0 ± 30.7 | (May - 2024, Aug - 2024, Sep - 2024, June - 2025) |
| 989001044609844 | 3 | 199.0 ± 10.6 | 76.7 ± 6.0 | (May - 2024, July - 2024, Aug - 2024) |
| 989001044609849 | 3 | 192.3 ± 23.0 | 71.0 ± 25.9 | (May - 2024, June - 2024, July - 2024) |
| 989001044609876 | 3 | 174.0 ± 10.4 | 45.3 ± 7.2 | (June - 2023, July - 2023, Aug - 2023) |
| 989001044609952 | 3 | 165.0 ± 0.0 | 34.3 ± 8.7 | (May - 2024, Aug - 2024, Sep - 2024) |
| 989001044609976 | 3 | 194.0 ± 11.1 | 73.0 ± 12.5 | (May - 2024, June - 2024, Sep - 2024) |
| 989001044609982 | 4 | 164.5 ± 5.1 | 42.2 ± 3.1 | (May - 2024, July - 2024, Aug - 2024, Sep - 2024) |
| 989001044610033 | 3 | 202.0 ± 32.5 | 82.7 ± 39.1 | (Aug - 2023, May - 2024, Aug - 2024) |
| 989001044610049 | 4 | 150.8 ± 14.4 | 33.2 ± 13.1 | (Aug - 2023, Sep - 2023, June - 2024, July - 2024) |

| **Table S2**. Metadata of individuals captured in Obenhoff Creek, total length (TL) and mass (g) expressed as: (mean ± SD) | | | | |
| --- | --- | --- | --- | --- |
| **Individual ID** | **n** | **TL (mm)** | **Mass (g)** | **Sampling Events (Month - Year)** |
| 900251000082543 | 5 | 225.4 ± 18.4 | 106.8 ± 32.0 | (July - 2024, Sep - 2024, May - 2025, June - 2025, Aug - 2025) |
| 900251000082558 | 4 | 200.5 ± 14.4 | 78.5 ± 17.1 | (July - 2024, May - 2025, June - 2025, Aug - 2025) |
| 900251000082564 | 3 | 167.0 ± 1.0 | 44.3 ± 3.1 | (July - 2024, Aug - 2024, Sep - 2024) |
| 900251000082577 | 4 | 154.8 ± 4.5 | 29.0 ± 3.4 | (July - 2024, Sep - 2024, May - 2025, June - 2025) |
| 900251000082581 | 3 | 212.7 ± 4.0 | 93.0 ± 7.9 | (July - 2024, May - 2025, June - 2025) |
| 900251000082584 | 4 | 199.2 ± 7.8 | 69.5 ± 10.3 | (July - 2024, Aug - 2024, Sep - 2024, May - 2025) |
| 900251000082622 | 3 | 156.7 ± 3.2 | 39.0 ± 5.3 | (June - 2024, July - 2024, Aug - 2024) |
| 900251000082637 | 3 | 194.7 ± 6.7 | 73.3 ± 3.8 | (June - 2024, July - 2024, June - 2025) |
| 900251000082643 | 5 | 137.8 ± 8.4 | 25.2 ± 3.3 | (June - 2024, July - 2024, Aug - 2024, Sep - 2024, May - 2025) |
| 900251000082677 | 8 | 174.2 ± 11.8 | 50.4 ± 8.7 | (June - 2024, Aug - 2024, Sep - 2024, May - 2025, June - 2025, July - 2025, Aug - 2025, Sep - 2025) |
| 900251000082690 | 4 | 137.0 ± 9.2 | 26.8 ± 5.1 | (June - 2024, July - 2024, May - 2025, June - 2025) |
| 900251000082706 | 5 | 159.6 ± 7.7 | 38.4 ± 4.7 | (June - 2024, July - 2024, Aug - 2024, May - 2025, June - 2025) |
| 900251000082717 | 7 | 190.4 ± 12.7 | 61.7 ± 12.9 | (June - 2024, July - 2024, Aug - 2024, Sep - 2024, May - 2025, June - 2025, Sep - 2025) |
| 900251000082729 | 3 | 136.3 ± 0.6 | 20.7 ± 1.5 | (July - 2024, Aug - 2024, Sep - 2024) |
| 900251000082735 | 8 | 174.6 ± 9.8 | 55.6 ± 13.5 | (July - 2024, Aug - 2024, Sep - 2024, May - 2025, June - 2025, July - 2025, Aug - 2025, Sep – 2025) |
| 900251000082754 | 3 | 151.0 ± 23.6 | 34.3 ± 16.7 | (Aug - 2024, May - 2025, Aug - 2025) |
| 900251000082772 | 3 | 137.3 ± 13.7 | 24.0 ± 8.2 | (Sep - 2024, June - 2025, Aug - 2025) |
| 900251000082780 | 3 | 135.7 ± 3.1 | 22.7 ± 5.5 | (July - 2024, Sep - 2024, June - 2025) |
| 900251000082794 | 5 | 132.2 ± 12.3 | 21.0 ± 6.5 | (Aug - 2024, Sep - 2024, May - 2025, Aug - 2025, Sep - 2025) |
| 900251000082832 | 4 | 135.5 ± 7.7 | 22.2 ± 3.9 | (May - 2025, June - 2025, Aug - 2025, Sep - 2025) |
| 900251000082846 | 3 | 144.7 ± 11.2 | 27.7 ± 6.7 | (May - 2025, June - 2025, Sep - 2025) |
| 900251000082872 | 3 | 140.7 ± 5.9 | 24.3 ± 4.0 | (May - 2025, July - 2025, Aug - 2025) |
| 900251000082896 | 3 | 139.7 ± 7.5 | 25.7 ± 6.0 | (May - 2025, June - 2025, July - 2025) |
| 900251000470039 | 3 | 142.3 ± 2.1 | 26.0 ± 0.0 | (July - 2025, Aug - 2025, Sep - 2025) |
| 900251000470116 | 3 | 155.7 ± 11.0 | 34.0 ± 7.2 | (May - 2025, June - 2025, Aug - 2025) |
| 900251000470172 | 3 | 155.7 ± 6.7 | 36.0 ± 4.4 | (June - 2025, Aug - 2025, Sep - 2025) |

| **Table S3**. Fatty acid composition of each individual (mean ± standard deviation) in the Salmon-Trout River. | | | | | | | | | | | | | | | | | | |
| --- | --- | --- | --- | --- | --- | --- | --- | --- | --- | --- | --- | --- | --- | --- | --- | --- | --- | --- |
| **Individual ID** | **n** | **C16:0** | **C16:1n-7** | **C17:0** | **C18:0** | **C18:1n-9** | **C18:1n-7** | **C18:2n-6** | **C18:3n-3** | **C20:0** | **C20:1** | **C20:2n-6** | **C20:3n-6** | **C20:4n-6** | **C20:4n-3** | **C20:5n-3** | **C22:5n-3** | **C22:6n-3** |
| 900251000082552 | 3 | 17.13 ± 4.16 | 2.88 ± 0.39 | 1.78 ± 0.65 | 5.54 ± 1.40 | 10.57 ± 2.75 | 4.25 ± 0.27 | 2.89 ± 0.58 | 1.94 ± 0.09 | 1.38 ± 0.57 | 3.82 ± 1.29 | 1.68 ± 0.29 | 1.51 ± 0.15 | 6.06 ± 2.03 | 0.61 ± 0.09 | 10.10 ± 3.67 | 2.64 ± 1.09 | 15.17 ± 7.35 |
| 900251000082554 | 3 | 21.45 ± 2.64 | 4.14 ± 1.58 | 1.41 ± 0.62 | 5.96 ± 1.57 | 13.09 ± 3.81 | 4.51 ± 1.21 | 2.88 ± 1.30 | 2.48 ± 1.40 | 1.04 ± 1.00 | 2.24 ± 1.10 | 0.83 ± 0.01 | 0.88 ± 0.11 | 4.09 ± 2.60 | 0.56 ± 0.16 | 11.40 ± 3.36 | 2.53 ± 2.05 | 13.36 ± 10.22 |
| 900251000082571 | 4 | 17.66 ± 1.50 | 3.59 ± 0.51 | 1.50 ± 0.21 | 5.14 ± 0.71 | 9.56 ± 0.63 | 4.49 ± 0.38 | 1.72 ± 0.13 | 1.04 ± 0.29 | 1.93 ± 1.90 | 3.40 ± 1.67 | 1.17 ± 0.19 | 1.05 ± 0.15 | 7.55 ± 2.43 | 0.51 ± 0.23 | 11.78 ± 3.67 | 2.87 ± 0.42 | 15.81 ± 4.26 |
| 900251000082645 | 3 | 19.25 ± 7.72 | 3.15 ± 0.58 | 1.54 ± 0.47 | 5.89 ± 1.35 | 13.43 ± 6.71 | 4.16 ± 0.64 | 3.56 ± 0.93 | 1.30 ± 0.32 | 0.69 ± 0.47 | 1.91 ± 0.73 | 1.12 ± 0.49 | 0.89 ± 0.27 | 6.85 ± 3.84 | 4.72 ± 7.12 | 6.50 ± 2.15 | 1.83 ± 1.10 | 14.84 ± 9.69 |
| 900251000082666 | 3 | 18.86 ± 1.63 | 4.81 ± 1.33 | 0.99 ± 0.17 | 5.27 ± 0.50 | 12.72 ± 1.76 | 5.17 ± 0.75 | 2.75 ± 0.38 | 1.73 ± 0.79 | 0.29 ± 0.07 | 1.52 ± 0.32 | 0.71 ± 0.22 | 0.70 ± 0.18 | 5.54 ± 1.51 | 0.64 ± 0.22 | 8.66 ± 3.09 | 2.84 ± 0.74 | 20.74 ± 4.30 |
| 900251000082822 | 3 | 17.18 ± 1.51 | 7.98 ± 1.38 | 1.08 ± 0.20 | 4.92 ± 0.23 | 11.27 ± 1.13 | 7.15 ± 2.12 | 2.23 ± 0.26 | 2.49 ± 0.53 | 0.40 ± 0.17 | 1.96 ± 0.34 | 0.68 ± 0.20 | 0.78 ± 0.10 | 4.09 ± 0.81 | 0.62 ± 0.01 | 12.01 ± 2.34 | 4.16 ± 1.09 | 14.25 ± 1.93 |
| 900251000082854 | 3 | 16.66 ± 3.15 | 3.44 ± 1.27 | 1.42 ± 0.46 | 4.64 ± 0.78 | 8.41 ± 1.76 | 4.12 ± 1.28 | 3.29 ± 1.15 | 2.04 ± 0.45 | 0.90 ± 0.32 | 2.18 ± 0.52 | 1.01 ± 0.28 | 1.28 ± 0.26 | 5.86 ± 1.85 | 0.63 ± 0.16 | 16.05 ± 2.14 | 2.94 ± 0.35 | 17.36 ± 2.62 |
| 900251000082887 | 3 | 16.51 ± 1.22 | 2.45 ± 0.51 | 1.46 ± 0.36 | 5.52 ± 1.18 | 11.24 ± 1.47 | 3.82 ± 0.20 | 2.94 ± 0.42 | 1.92 ± 0.26 | 0.78 ± 0.44 | 2.77 ± 0.51 | 1.45 ± 0.41 | 1.28 ± 0.24 | 7.80 ± 0.39 | 0.47 ± 0.03 | 8.79 ± 0.99 | 2.76 ± 0.23 | 19.73 ± 4.25 |
| 989001044609756 | 4 | 17.91 ± 3.39 | 3.45 ± 0.62 | 1.39 ± 0.27 | 6.02 ± 1.45 | 11.79 ± 3.67 | 5.40 ± 1.30 | 2.85 ± 0.53 | 1.87 ± 0.10 | 0.72 ± 0.35 | 2.49 ± 1.10 | 1.15 ± 0.40 | 0.91 ± 0.31 | 5.90 ± 1.33 | 0.67 ± 0.04 | 9.50 ± 3.61 | 3.03 ± 0.83 | 17.93 ± 4.21 |
| 989001044609767 | 3 | 19.48 ± 3.58 | 5.40 ± 1.48 | 1.11 ± 0.18 | 6.26 ± 0.43 | 12.55 ± 2.86 | 6.55 ± 0.62 | 2.61 ± 1.35 | 1.60 ± 1.08 | 0.68 ± 0.39 | 2.45 ± 0.48 | 0.91 ± 0.45 | 0.77 ± 0.29 | 4.45 ± 2.36 | 3.64 ± 5.06 | 8.21 ± 2.03 | 2.25 ± 0.60 | 14.29 ± 5.05 |
| 989001044609784 | 3 | 19.37 ± 8.56 | 2.81 ± 1.16 | 1.39 ± 0.39 | 5.82 ± 2.29 | 14.47 ± 5.43 | 4.05 ± 0.89 | 3.58 ± 0.19 | 1.93 ± 0.91 | 0.86 ± 0.38 | 2.01 ± 1.13 | 1.13 ± 0.09 | 0.94 ± 0.01 | 6.14 ± 3.78 | 0.91 ± 0.47 | 7.67 ± 3.76 | 2.01 ± 1.24 | 15.86 ± 11.04 |
| 989001044609818 | 6 | 18.38 ± 4.89 | 7.27 ± 3.61 | 1.12 ± 0.23 | 5.25 ± 1.10 | 11.03 ± 1.02 | 7.44 ± 2.14 | 2.52 ± 0.88 | 2.05 ± 1.15 | 0.54 ± 0.30 | 2.30 ± 0.88 | 0.94 ± 0.39 | 0.70 ± 0.36 | 3.93 ± 1.77 | 0.83 ± 0.28 | 10.37 ± 2.27 | 3.82 ± 1.22 | 15.15 ± 5.75 |
| 989001044609841 | 4 | 20.88 ± 5.46 | 4.72 ± 1.57 | 1.32 ± 0.46 | 5.88 ± 2.25 | 12.86 ± 3.79 | 5.05 ± 1.57 | 1.97 ± 0.20 | 1.46 ± 0.63 | 0.48 ± 0.37 | 1.98 ± 0.93 | 0.71 ± 0.31 | 0.73 ± 0.35 | 4.67 ± 0.74 | 0.55 ± 0.29 | 9.84 ± 5.09 | 2.74 ± 1.06 | 17.37 ± 6.64 |
| 989001044609844 | 3 | 22.01 ± 1.28 | 4.97 ± 4.69 | 1.32 ± 0.39 | 5.83 ± 0.45 | 11.56 ± 1.09 | 5.51 ± 2.50 | 3.44 ± 1.47 | 1.71 ± 0.29 | 0.42 ± 0.14 | 1.60 ± 0.43 | 1.21 ± 0.81 | 0.84 ± 0.41 | 6.42 ± 3.56 | 0.49 ± 0.11 | 8.96 ± 3.15 | 2.42 ± 0.24 | 14.23 ± 1.91 |
| 989001044609849 | 3 | 21.84 ± 3.85 | 6.07 ± 2.58 | 1.59 ± 0.29 | 7.26 ± 0.56 | 17.94 ± 5.80 | 6.74 ± 2.17 | 3.86 ± 1.79 | 1.54 ± 0.82 | 0.57 ± 0.04 | 2.67 ± 1.01 | 1.17 ± 0.42 | 0.75 ± 0.31 | 4.35 ± 2.78 | 0.52 ± 0.20 | 4.59 ± 2.71 | 1.56 ± 1.09 | 9.12 ± 7.10 |
| 989001044609876 | 3 | 31.38 ± 6.79 | 5.23 ± 2.36 | 1.96 ± 0.58 | 8.49 ± 1.75 | 15.35 ± 4.87 | 5.87 ± 0.51 | 3.39 ± 0.60 | 1.45 ± 0.44 | 0.62 ± 0.27 | 4.00 ± 3.70 | 0.74 ± 0.17 | 0.49 ± 0.16 | 1.66 ± 1.33 | 0.51 ± 0.17 | 2.02 ± 0.97 | 0.58 ± 0.34 | 4.93 ± 4.44 |
| 989001044609952 | 3 | 20.66 ± 3.60 | 4.84 ± 3.07 | 1.32 ± 0.47 | 6.85 ± 1.32 | 16.19 ± 10.85 | 4.47 ± 2.52 | 2.11 ± 0.44 | 0.90 ± 0.33 | 0.39 ± 0.17 | 2.03 ± 1.25 | 0.70 ± 0.19 | 0.72 ± 0.32 | 6.90 ± 4.59 | 0.58 ± 0.46 | 5.40 ± 3.55 | 1.80 ± 1.28 | 16.56 ± 12.19 |
| 989001044609976 | 3 | 22.84 ± 5.29 | 6.49 ± 4.31 | 1.03 ± 0.06 | 7.18 ± 0.84 | 13.53 ± 1.96 | 7.48 ± 2.64 | 2.63 ± 1.35 | 1.33 ± 0.45 | 0.71 ± 0.35 | 2.34 ± 0.27 | 0.82 ± 0.43 | 0.56 ± 0.32 | 3.70 ± 2.10 | 1.19 ± 1.07 | 8.03 ± 2.37 | 1.85 ± 0.94 | 11.71 ± 6.81 |
| 989001044609982 | 4 | 19.09 ± 6.87 | 4.57 ± 1.54 | 1.25 ± 0.19 | 6.28 ± 0.99 | 11.62 ± 3.60 | 5.62 ± 0.91 | 2.35 ± 0.63 | 1.61 ± 0.37 | 0.74 ± 0.43 | 1.94 ± 0.99 | 0.93 ± 0.23 | 0.78 ± 0.22 | 4.98 ± 2.25 | 0.72 ± 0.14 | 9.69 ± 6.27 | 3.39 ± 1.21 | 16.35 ± 9.42 |
| 989001044610033 | 3 | 22.06 ± 6.16 | 4.85 ± 0.64 | 1.58 ± 0.27 | 6.89 ± 2.34 | 16.63 ± 6.63 | 5.89 ± 0.93 | 2.69 ± 0.79 | 1.08 ± 0.59 | 0.61 ± 0.61 | 2.09 ± 0.71 | 0.89 ± 0.17 | 0.53 ± 0.08 | 5.38 ± 4.09 | 0.73 ± 0.60 | 6.57 ± 3.03 | 1.85 ± 1.42 | 11.77 ± 9.53 |
| 989001044610049 | 4 | 28.12 ± 7.05 | 4.22 ± 1.10 | 1.11 ± 0.13 | 7.92 ± 0.91 | 17.01 ± 6.49 | 5.20 ± 0.75 | 3.01 ± 0.50 | 1.43 ± 0.25 | 0.64 ± 0.29 | 1.61 ± 0.21 | 0.84 ± 0.36 | 0.56 ± 0.13 | 3.45 ± 2.90 | 1.46 ± 1.68 | 5.79 ± 3.21 | 1.36 ± 1.05 | 10.60 ± 7.87 |

| **Table S4**. Fatty acid composition of each individual (mean ± standard deviation) in the Obenhoff Creek. | | | | | | | | | | | | | | | | | | |
| --- | --- | --- | --- | --- | --- | --- | --- | --- | --- | --- | --- | --- | --- | --- | --- | --- | --- | --- |
| **Individual ID** | **n** | **C16:0** | **C16:1n-7** | **C17:0** | **C18:0** | **C18:1n-9** | **C18:1n-7** | **C18:2n-6** | **C18:3n-3** | **C20:0** | **C20:1** | **C20:2n-6** | **C20:3n-6** | **C20:4n-6** | **C20:4n-3** | **C20:5n-3** | **C22:5n-3** | **C22:6n-3** |
| 900251000082543 | 5 | 20.55 ± 3.95 | 3.64 ± 0.64 | 1.23 ± 0.24 | 5.45 ± 1.13 | 11.18 ± 3.60 | 4.74 ± 1.02 | 2.80 ± 0.80 | 1.43 ± 0.27 | 1.01 ± 0.73 | 2.49 ± 0.96 | 0.98 ± 0.28 | 1.00 ± 0.39 | 5.30 ± 0.95 | 0.59 ± 0.11 | 9.72 ± 4.71 | 2.19 ± 0.75 | 18.54 ± 3.70 |
| 900251000082558 | 4 | 18.89 ± 3.70 | 4.11 ± 2.29 | 1.66 ± 0.61 | 5.96 ± 1.52 | 10.32 ± 2.95 | 4.41 ± 1.24 | 2.99 ± 0.74 | 2.12 ± 0.52 | 1.21 ± 0.46 | 3.03 ± 1.92 | 1.22 ± 0.54 | 1.21 ± 0.45 | 4.71 ± 1.51 | 0.56 ± 0.10 | 12.27 ± 4.74 | 2.35 ± 0.96 | 14.68 ± 8.41 |
| 900251000082564 | 3 | 25.00 ± 7.75 | 5.71 ± 2.86 | 1.12 ± 0.05 | 6.07 ± 1.14 | 10.87 ± 0.98 | 4.70 ± 0.51 | 1.93 ± 0.72 | 1.35 ± 0.44 | 0.73 ± 0.32 | 1.48 ± 0.44 | 0.61 ± 0.02 | 0.51 ± 0.03 | 3.64 ± 1.82 | 4.08 ± 5.66 | 8.42 ± 2.70 | 1.78 ± 0.81 | 14.85 ± 8.09 |
| 900251000082577 | 4 | 22.44 ± 5.80 | 4.54 ± 2.43 | 1.90 ± 0.16 | 6.58 ± 1.78 | 9.68 ± 3.49 | 4.20 ± 0.77 | 3.37 ± 1.58 | 1.54 ± 0.64 | 1.56 ± 1.01 | 3.06 ± 0.97 | 1.41 ± 0.48 | 1.52 ± 0.53 | 5.01 ± 1.93 | 0.95 ± 0.92 | 9.74 ± 5.46 | 1.59 ± 0.63 | 11.06 ± 5.61 |
| 900251000082581 | 3 | 20.79 ± 4.76 | 4.03 ± 1.29 | 1.42 ± 0.46 | 5.64 ± 1.67 | 10.34 ± 3.60 | 4.82 ± 1.37 | 2.59 ± 0.64 | 1.51 ± 0.34 | 1.04 ± 0.69 | 2.83 ± 1.66 | 1.17 ± 0.41 | 1.06 ± 0.21 | 4.39 ± 1.70 | 0.42 ± 0.05 | 11.80 ± 3.34 | 2.58 ± 1.41 | 15.36 ± 10.82 |
| 900251000082584 | 4 | 19.20 ± 1.49 | 4.41 ± 1.03 | 1.16 ± 0.17 | 6.57 ± 1.38 | 11.54 ± 3.59 | 5.93 ± 1.58 | 3.04 ± 0.66 | 2.37 ± 0.45 | 0.61 ± 0.53 | 2.10 ± 0.83 | 1.20 ± 0.19 | 0.85 ± 0.47 | 4.79 ± 1.45 | 0.75 ± 0.24 | 8.69 ± 3.80 | 3.45 ± 0.28 | 17.48 ± 2.27 |
| 900251000082622 | 3 | 19.05 ± 0.72 | 6.85 ± 1.08 | 1.06 ± 0.36 | 6.88 ± 1.07 | 15.65 ± 3.13 | 7.51 ± 0.68 | 3.82 ± 0.48 | 2.07 ± 0.28 | 0.60 ± 0.14 | 2.12 ± 1.03 | 0.95 ± 0.24 | 0.55 ± 0.13 | 2.80 ± 0.29 | 2.12 ± 2.40 | 7.17 ± 1.90 | 2.26 ± 0.83 | 12.62 ± 2.81 |
| 900251000082637 | 3 | 19.67 ± 6.48 | 4.85 ± 1.58 | 1.62 ± 0.18 | 6.16 ± 2.18 | 14.54 ± 4.52 | 5.39 ± 1.38 | 5.32 ± 0.48 | 2.02 ± 0.40 | 0.81 ± 0.67 | 2.31 ± 0.55 | 1.18 ± 0.18 | 1.02 ± 0.35 | 3.55 ± 0.64 | 0.68 ± 0.10 | 10.28 ± 8.68 | 2.05 ± 0.58 | 11.45 ± 2.22 |
| 900251000082643 | 5 | 23.01 ± 4.08 | 5.17 ± 1.23 | 1.49 ± 0.54 | 6.84 ± 2.04 | 11.99 ± 3.39 | 5.69 ± 1.64 | 3.76 ± 0.87 | 1.58 ± 0.76 | 0.77 ± 0.25 | 2.01 ± 0.60 | 0.98 ± 0.21 | 0.84 ± 0.18 | 4.01 ± 1.23 | 0.83 ± 0.37 | 8.84 ± 5.18 | 1.69 ± 0.67 | 13.43 ± 5.53 |
| 900251000082677 | 8 | 16.44 ± 4.91 | 4.59 ± 1.67 | 1.52 ± 0.29 | 5.89 ± 2.00 | 12.61 ± 3.93 | 5.50 ± 1.81 | 4.10 ± 1.27 | 1.34 ± 0.41 | 1.68 ± 2.14 | 3.52 ± 2.25 | 1.58 ± 0.49 | 1.29 ± 0.63 | 5.19 ± 1.35 | 1.61 ± 3.09 | 10.07 ± 4.51 | 2.67 ± 0.81 | 11.36 ± 2.24 |
| 900251000082690 | 4 | 19.77 ± 1.84 | 4.56 ± 1.59 | 1.30 ± 0.33 | 5.77 ± 1.47 | 10.17 ± 4.93 | 5.54 ± 1.25 | 2.41 ± 0.55 | 1.86 ± 0.63 | 1.19 ± 0.90 | 2.80 ± 1.28 | 1.10 ± 0.48 | 0.96 ± 0.51 | 4.46 ± 0.34 | 0.54 ± 0.17 | 11.48 ± 5.28 | 2.75 ± 0.12 | 16.37 ± 5.35 |
| 900251000082706 | 5 | 19.93 ± 3.57 | 4.38 ± 1.39 | 1.12 ± 0.54 | 5.56 ± 1.59 | 10.81 ± 3.91 | 5.54 ± 1.38 | 3.88 ± 1.18 | 1.52 ± 0.49 | 1.11 ± 0.66 | 2.54 ± 0.87 | 1.36 ± 0.44 | 1.12 ± 0.37 | 4.23 ± 0.76 | 1.56 ± 1.37 | 12.66 ± 7.21 | 2.51 ± 0.89 | 13.13 ± 4.05 |
| 900251000082717 | 7 | 20.31 ± 3.69 | 4.21 ± 1.30 | 1.28 ± 0.41 | 6.04 ± 1.35 | 10.31 ± 2.89 | 5.03 ± 1.16 | 3.06 ± 0.57 | 1.58 ± 0.38 | 0.81 ± 0.43 | 1.91 ± 0.87 | 0.93 ± 0.42 | 0.80 ± 0.45 | 4.97 ± 1.53 | 1.07 ± 1.37 | 12.16 ± 4.23 | 2.78 ± 0.79 | 16.51 ± 3.32 |
| 900251000082729 | 3 | 29.90 ± 8.24 | 6.75 ± 1.35 | 0.94 ± 0.10 | 6.53 ± 1.41 | 9.89 ± 2.41 | 5.66 ± 1.09 | 1.47 ± 0.90 | 1.06 ± 0.54 | 1.05 ± 0.73 | 2.47 ± 1.58 | 0.61 ± 0.06 | 0.60 ± 0.20 | 1.87 ± 1.00 | 5.00 ± 7.35 | 6.97 ± 3.53 | 1.33 ± 0.48 | 8.78 ± 5.54 |
| 900251000082735 | 8 | 17.57 ± 2.44 | 4.71 ± 1.05 | 1.42 ± 0.20 | 6.02 ± 1.14 | 11.66 ± 2.74 | 5.84 ± 1.39 | 2.80 ± 0.82 | 1.77 ± 0.65 | 1.00 ± 0.64 | 2.87 ± 0.91 | 1.32 ± 0.27 | 1.11 ± 0.41 | 5.19 ± 0.92 | 0.68 ± 0.26 | 10.61 ± 5.62 | 3.33 ± 0.74 | 14.52 ± 2.32 |
| 900251000082754 | 3 | 15.91 ± 1.92 | 3.36 ± 0.58 | 1.74 ± 0.24 | 4.94 ± 0.85 | 9.21 ± 2.91 | 4.26 ± 0.41 | 2.36 ± 0.65 | 1.53 ± 0.76 | 1.80 ± 1.25 | 3.92 ± 1.81 | 1.45 ± 0.53 | 1.26 ± 0.38 | 5.48 ± 4.07 | 0.46 ± 0.15 | 18.04 ± 10.49 | 2.45 ± 1.50 | 12.03 ± 9.43 |
| 900251000082772 | 3 | 11.98 ± 2.16 | 2.51 ± 0.46 | 2.09 ± 0.57 | 3.74 ± 0.72 | 9.17 ± 4.42 | 3.09 ± 0.54 | 3.45 ± 1.25 | 1.96 ± 0.25 | 2.98 ± 1.41 | 4.89 ± 1.00 | 2.28 ± 0.66 | 2.04 ± 0.50 | 6.41 ± 1.70 | 0.62 ± 0.09 | 16.59 ± 8.41 | 2.24 ± 0.61 | 10.94 ± 3.64 |
| 900251000082780 | 3 | 18.65 ± 8.05 | 2.73 ± 0.72 | 1.78 ± 0.51 | 5.79 ± 2.25 | 8.27 ± 4.49 | 4.05 ± 1.93 | 2.87 ± 0.65 | 2.00 ± 0.28 | 1.91 ± 1.20 | 5.20 ± 5.39 | 1.72 ± 1.05 | 1.27 ± 0.75 | 4.44 ± 0.67 | 0.69 ± 0.14 | 12.88 ± 7.50 | 2.33 ± 1.15 | 11.78 ± 6.81 |
| 900251000082794 | 5 | 16.24 ± 6.19 | 3.30 ± 1.79 | 1.64 ± 0.14 | 5.14 ± 1.58 | 8.76 ± 3.70 | 4.06 ± 1.41 | 2.38 ± 1.03 | 1.42 ± 0.45 | 2.02 ± 2.07 | 2.90 ± 0.71 | 1.57 ± 0.37 | 1.60 ± 0.64 | 5.59 ± 2.96 | 0.48 ± 0.14 | 18.45 ± 17.07 | 2.41 ± 0.91 | 11.85 ± 6.38 |
| 900251000082832 | 4 | 16.14 ± 1.89 | 2.70 ± 1.25 | 1.71 ± 0.57 | 4.96 ± 0.47 | 7.31 ± 1.61 | 4.06 ± 1.21 | 2.60 ± 0.43 | 2.25 ± 0.79 | 1.39 ± 0.34 | 3.11 ± 0.77 | 1.67 ± 0.40 | 1.44 ± 0.40 | 6.21 ± 1.80 | 0.63 ± 0.24 | 14.19 ± 1.95 | 2.98 ± 0.17 | 16.71 ± 2.82 |
| 900251000082846 | 3 | 14.30 ± 4.39 | 3.13 ± 0.79 | 1.77 ± 0.24 | 4.74 ± 1.83 | 9.17 ± 4.53 | 4.45 ± 1.68 | 3.27 ± 0.72 | 1.92 ± 0.67 | 1.52 ± 1.02 | 5.00 ± 3.23 | 2.03 ± 0.58 | 2.14 ± 1.25 | 5.33 ± 1.12 | 0.75 ± 0.46 | 11.67 ± 4.83 | 3.47 ± 0.63 | 13.85 ± 1.81 |
| 900251000082872 | 3 | 15.74 ± 2.98 | 3.40 ± 1.24 | 1.94 ± 0.94 | 5.28 ± 1.47 | 8.42 ± 3.27 | 4.38 ± 0.72 | 2.08 ± 0.38 | 1.46 ± 0.57 | 2.63 ± 0.96 | 5.12 ± 1.69 | 1.50 ± 0.27 | 1.46 ± 0.29 | 4.87 ± 0.58 | 0.51 ± 0.11 | 16.07 ± 6.25 | 2.29 ± 0.70 | 10.29 ± 6.03 |
| 900251000082896 | 3 | 17.11 ± 1.82 | 2.91 ± 0.52 | 1.94 ± 0.56 | 5.11 ± 1.04 | 7.65 ± 1.37 | 4.05 ± 0.15 | 3.26 ± 0.53 | 1.45 ± 0.15 | 1.72 ± 0.68 | 3.29 ± 0.79 | 1.66 ± 0.63 | 1.57 ± 0.24 | 5.79 ± 1.53 | 0.51 ± 0.12 | 15.18 ± 6.75 | 2.14 ± 0.15 | 15.15 ± 2.76 |
| 900251000470039 | 3 | 13.93 ± 0.77 | 3.69 ± 0.12 | 1.56 ± 0.12 | 5.28 ± 0.25 | 12.41 ± 1.65 | 4.91 ± 0.41 | 3.86 ± 1.15 | 2.23 ± 1.01 | 1.09 ± 0.44 | 3.44 ± 0.78 | 2.01 ± 0.60 | 1.66 ± 0.40 | 7.11 ± 0.29 | 0.62 ± 0.12 | 8.61 ± 4.53 | 3.51 ± 0.66 | 14.66 ± 0.44 |
| 900251000470116 | 3 | 15.05 ± 0.79 | 2.94 ± 0.26 | 1.91 ± 0.14 | 5.12 ± 0.52 | 9.22 ± 1.75 | 4.17 ± 0.90 | 3.89 ± 1.88 | 2.12 ± 1.08 | 1.88 ± 0.26 | 3.74 ± 0.52 | 1.74 ± 0.27 | 1.75 ± 0.25 | 6.04 ± 1.23 | 0.52 ± 0.15 | 11.65 ± 1.79 | 2.50 ± 0.32 | 15.61 ± 2.72 |
| 900251000470172 | 3 | 14.30 ± 1.43 | 2.19 ± 0.28 | 1.52 ± 0.29 | 4.51 ± 0.45 | 8.42 ± 0.85 | 3.61 ± 0.13 | 3.33 ± 0.79 | 1.74 ± 0.55 | 1.41 ± 0.33 | 3.33 ± 0.51 | 1.58 ± 0.25 | 1.49 ± 0.10 | 6.98 ± 1.57 | 0.57 ± 0.15 | 16.76 ± 3.57 | 2.57 ± 0.25 | 16.13 ± 4.14 |

| **Table S5**. Linear model results from stepwise comparison for Obenhoff Creek, with change in total niche width (ΔTNW) as the response variable. | | | | | | | |
| --- | --- | --- | --- | --- | --- | --- | --- |
| Model | Predictor | Estimate | Std. Error | t value | p | Adj R² | AIC |
| M1 | (Intercept) | -6.39E-03 | 2.30E-02 | -0.278 | 0.783 | 0.237 | -155.829 |
|  | Niche Position | 2.47E-02 | 7.55E-03 | 3.275 | 0.004 |  |  |
|  | Orientation | -2.36E-06 | 3.74E-05 | -0.063 | 0.950 |  |  |
|  | Eccentricity | -1.16E-02 | 2.23E-02 | -0.521 | 0.608 |  |  |
|  | RINI | 3.47E-03 | 8.84E-03 | 0.392 | 0.699 |  |  |
| M2 | (Intercept) | -6.55E-03 | 2.23E-02 | -0.294 | 7.72E-01 | 0.271 | -157.824 |
|  | Niche Position | 2.46E-02 | 7.15E-03 | 3.44 | 0.002 |  |  |
|  | Eccentricity | -1.17E-02 | 2.18E-02 | -0.537 | 0.596 |  |  |
|  | RINI | 3.60E-03 | 8.37E-03 | 0.43 | 0.671 |  |  |
| M3 | (Intercept) | -1.41E-03 | 1.85E-02 | -0.076 | 9.40E-01 | 0.297 | -159.606 |
|  | Niche Position | 2.43E-02 | 6.99E-03 | 3.476 | 0.002 |  |  |
|  | Eccentricity | -1.53E-02 | 1.97E-02 | -0.778 | 0.444 |  |  |
| M4 | (Intercept) | -1.54E-02 | 4.02E-03 | -3.839 | 7.91E-04 | 0.309 | -160.931 |
|  | Niche Position | 2.42E-02 | 6.93E-03 | 3.487 | 0.002 |  |  |

| **Table S6**. Linear model results from stepwise comparison for Salmon-Trout River, with change in total niche width (ΔTNW) as the response variable. | | | | | | | |
| --- | --- | --- | --- | --- | --- | --- | --- |
| Model | Predictor | Estimate | Std. Error | t value | p | Adj R² | AIC |
| M1 | (Intercept) | -3.89E-03 | 3.59E-02 | -0.109 | 0.915 | 0.322 | -115.511 |
|  | Niche Position | 1.98E-02 | 8.12E-03 | 2.433 | 0.027 |  |  |
|  | Orientation | -2.96E-05 | 5.37E-05 | -0.551 | 0.589 |  |  |
|  | Eccentricity | -2.28E-02 | 3.54E-02 | -0.645 | 0.528 |  |  |
|  | RINI | -2.91E-02 | 1.26E-02 | -2.305 | 0.035 |  |  |
| M2 | (Intercept) | -1.08E-02 | 3.29E-02 | -0.329 | 7.46E-01 | 0.349 | -117.116 |
|  | Niche Position | 1.99E-02 | 7.95E-03 | 2.508 | 0.023 |  |  |
|  | Eccentricity | -1.74E-02 | 3.33E-02 | -0.523 | 0.608 |  |  |
|  | RINI | -3.10E-01 | 1.19E-02 | -2.602 | 0.019 |  |  |
| M3 | (Intercept) | -2.76E-02 | 6.93E-03 | -3.98 | 8.77E-04 | 0.376 | -118.782 |
|  | Niche Position | 1.96E-02 | 7.76E-03 | 2.528 | 0.021 |  |  |
|  | RINI | -2.92E-02 | 1.12E-02 | -2.615 | 0.018 |  |  |

| **Table S7**. Linear regression results of the contribution to TNW of an individual and the average fatty acid content for Salmon-Trout River. Rows in bold indicate significant relationships. | | | | |
| --- | --- | --- | --- | --- |
| Predictor | Estimate | Std. Error | t-value | p |
| **16:0** | **0.0025** | **0.0008** | **2.9835** | **0.0076** |
| 16:1n-7 | -0.0023 | 0.0025 | -0.9349 | 0.3616 |
| **17:0** | **0.0308** | **0.0131** | **2.3443** | **0.0301** |
| **18:0** | **0.0099** | **0.0030** | **3.3035** | **0.0037** |
| **18:1n-9** | **0.0044** | **0.0011** | **4.0274** | **0.0007** |
| 18:1n-7 | -0.0037 | 0.0031 | -1.1992 | 0.2452 |
| 18:2n-6 | 0.0086 | 0.0063 | 1.3674 | 0.1875 |
| **18:3n-3** | **-0.0215** | **0.0072** | **-2.9838** | **0.0076** |
| 20:0 | -0.0001 | 0.0101 | -0.0057 | 0.9955 |
| 20:1 | 0.0014 | 0.0055 | 0.2505 | 0.8049 |
| 20:2n-6 | -0.0043 | 0.0142 | -0.3049 | 0.7637 |
| 20:3n-6 | -0.0151 | 0.0138 | -1.0948 | 0.2873 |
| 20:4n-3 | -0.0005 | 0.0025 | -0.1932 | 0.8489 |
| 20:4n-6 | 0.0030 | 0.0033 | 0.8887 | 0.3853 |
| **20:5n-3** | **-0.0032** | **0.0010** | **-3.2234** | **0.0045** |
| **22:5n-3** | **-0.0151** | **0.0027** | **-5.5038** | **0.00003** |
| **22:6n-3** | **-0.0024** | **0.0009** | **-2.6861** | **0.0146** |
| MUFA | 0.0013 | 0.0009 | 1.4101 | 0.1747 |
| **SAFA** | **0.0021** | **0.0006** | **3.4937** | **0.0024** |
| **PUFA** | **-0.0011** | **0.0004** | **-2.789** | **0.0117** |

| **Table S8**. Linear regression results of the contribution to TNW of an individual and the average fatty acid content for Obenhoff Creek. Rows in bold indicate significant relationships. | | | | |
| --- | --- | --- | --- | --- |
| Predictor | Estimate | Std. Error | t-value | p |
| **16:0** | **0.0014** | **0.0006** | **2.4229** | **0.0233** |
| 16:1n-7 | 0.0029 | 0.0020 | 1.4507 | 0.1598 |
| 17:0 | -0.0033 | 0.0080 | -0.4128 | 0.6834 |
| 18:0 | 0.0013 | 0.0033 | 0.3891 | 0.7007 |
| 18:1n-7 | -0.0010 | 0.0012 | -0.8233 | 0.4184 |
| 18:1n-9 | -0.0016 | 0.0027 | -0.5865 | 0.5630 |
| **18:2n-6** | **-0.0066** | **0.0028** | **-2.3678** | **0.0263** |
| **18:3n-3** | **-0.0169** | **0.0065** | **-2.6230** | **0.0149** |
| 20:0 | 0.0038 | 0.0041 | 0.9350 | 0.3591 |
| 20:1 | 0.0006 | 0.0024 | 0.2392 | 0.8130 |
| 20:2n-6 | -0.0067 | 0.0059 | -1.1445 | 0.2637 |
| 20:3n-6 | -0.0041 | 0.0058 | -0.6977 | 0.4921 |
| **20:4n-3** | **-0.0047** | **0.0019** | **-2.5250** | **0.0186** |
| **20:4n-6** | **0.0063** | **0.0019** | **3.3950** | **0.0024** |
| 20:5n-3 | 0.0002 | 0.0008 | 0.2466 | 0.8073 |
| **22:5n-3** | **-0.0130** | **0.0035** | **-3.6788** | **0.0012** |
| **22:6n-3** | **-0.0032** | **0.0008** | **-4.0416** | **0.0005** |
| MUFA | -0.0004 | 0.0008 | 0.5138 | 0.6121 |
| **SAFA** | **0.0017** | **0.0005** | **2.9850** | **0.0064** |
| **PUFA** | **-0.0009** | **0.0004** | **-2.150** | **0.0419** |

| **Table S9**. Population data of Salmon-Trout River and Obenhoff Creek, total length (TL) and mass (g) and fatty acids (percent of total fatty acids) expressed as: (mean ± SD) | | | | |
| --- | --- | --- | --- | --- |
|  | Salmon-Trout River | |  | Obenhoff Creek |
|  | Brook Trout | Rainbow Trout |  | Brook Trout |
| Abundance (n) | 71 | 57 |  | 105 |
| Length (mm) | 191 ± 39.1 | 160 ± 44.4 |  | 164 ± 28.3 |
| Mass (g) | 74.9 ± 50.3 | 50.2 ± 44.7 |  | 44.9 ± 25.5 |
|  |  |  |  |  |
| C16:0 | 20.35 ± 5.47 | 26.55 ± 6.66 |  | 18.60 ± 5.03 |
| C16:1n-7 | 4.71 ± 2.35 | 5.39 ± 2.79 |  | 4.12 ± 1.61 |
| C17:0 | 1.35 ± 0.38 | 1.26 ± 0.42 |  | 1.51 ± 0.44 |
| C18:0 | 6.10 ± 1.47 | 7.49 ± 1.81 |  | 5.71 ± 1.45 |
| C18:1n-9 | 12.88 ± 4.40 | 9.61± 2.91 |  | 10.53 ± 3.44 |
| C18:1n-7 | 5.45 ± 1.71 | 4.75 ± 1.79 |  | 4.93 ± 1.40 |
| C18:2n-6 | 2.78 ± 0.91 | 2.57 ± 1.02 |  | 3.13 ± 1.10 |
| C18:3n-3 | 1.67 ± 0.70 | 2.01 ± 0.98 |  | 1.71 ± 0.59 |
| C20:0 | 0.74 ± 0.63 | 0.88 ± 0.64 |  | 1.32 ± 1.05 |
| C20:1 | 2.34 ± 1.17 | 1.57 ± 0.90 |  | 3.03 ± 1.63 |
| C20:2n-6 | 0.99 ± 0.39 | 0.68 ± 0.37 |  | 1.35 ± 0.53 |
| C20:3n-6 | 0.83 ± 0.33 | 0.67± 0.39 |  | 1.21 ± 0.56 |
| C20:4n-6 | 5.18 ± 2.58 | 3.21 ± 1.87 |  | 4.95 ± 1.69 |
| C20:4n-3 | 1.00 ± 1.85 | 1.16 ± 1.82 |  | 1.04 ± 1.89 |
| C20:5n-3 | 8.78 ± 4.02 | 10.04 ± 4.50 |  | 11.82 ± 6.42 |
| C22:5n-3 | 2.51 ± 1.22 | 1.62 ± 0.78 |  | 2.52 ± 0.86 |
| C22:6n-3 | 14.72 ± 6.85 | 13.30 ± 7.42 |  | 13.94 ± 4.88 |
